# Kaempferol Reduces Matrix Metalloproteinase-2 Expression by Down-Regulating ERK1/2 and the Activator Protein-1 Signaling Pathways in Oral Cancer Cells

**DOI:** 10.1371/journal.pone.0080883

**Published:** 2013-11-20

**Authors:** Chiao-Wen Lin, Pei-Ni Chen, Mu-Kuan Chen, Wei-En Yang, Chih-Hsin Tang, Shun-Fa Yang, Yih-Shou Hsieh

**Affiliations:** 1 Institute of Oral Sciences, Chung Shan Medical University, Taichung, Taiwan; 2 Department of Dentistry, Chung Shan Medical University Hospital, Taichung, Taiwan; 3 Institute of Biochemistry and Biotechnology, Chung Shan Medical University, Taichung, Taiwan; 4 Department of Clinical Laboratory, Chung Shan Medical University Hospital, Taichung, Taiwan; 5 Department of Otorhinolaryngology-Head and Neck Surgery, Changhua Christian Hospital, Changhua, Taiwan; 6 Institute of Medicine, Chung Shan Medical University, Taichung, Taiwan; 7 Graduate Institute of Basic Medical Science, China Medical University, Taichung, Taiwan; 8 Department of Biotechnology, College of Health Science, Asia University, Taichung, Taiwan; 9 Department of Medical Research, Chung Shan Medical University Hospital, Taichung, Taiwan; The University of Hong Kong, China

## Abstract

**Background:**

Kaempferol has been proposed as a potential drug for cancer chemoprevention and treatment because it is a natural polyphenol contained in plant-based foods. Recent studies have demonstrated that kaempferol protects against cardiovascular disease and cancer. Based on this finding, we investigated the mechanisms by which kaempferol produces the anti-metastatic effect in human tongue squamous cell carcinoma SCC4 cells.

**Methodology/Principal Findings:**

In this study, we provided molecular evidence associated with the anti-metastatic effect of kaempferol by demonstrating a substantial suppression of SCC4 cell migration and invasion. This effect was associated with reduced expressions of MMP-2 and TIMP-2 mRNA and protein levels. Analysis of the transcriptional regulation indicated that kaempferol inhibited MMP-2 transcription by suppressing c-Jun activity. Kaempferol also produced an inhibitory effect on the phosphorylation of ERK1/2.

**Conclusions:**

These findings provide new insights into the molecular mechanisms involved in the anti-metastatic effect of kaempferol, and are valuable in the prevention of oral cancer metastasis.

## Introduction

Oral squamous cell carcinomas (OSCC) are the sixth most common malignancy worldwide and the fourth leading cause of cancer deaths among men in Taiwan [1]. Surgery and radiation therapy are a critical treatment modality in the early stage of OSCC [2]. Despite advances in therapy, OSCC is still characterized as recurrent and metastasis to regional cervical lymph nodes, which produces poor patient prognoses. The 5-year survival rate of cancer patients is less than 50%, but the chances of early diagnosis and prevention of this disease is likely to increase if the molecular mechanism is identified [3]. Although the cause of OSCC is multifactorial, metastasis, which is resistant to conventional therapies, is likely to be the main cause of death [4].

Degradation of basement membranes and stromal extracellular matrix (ECM) components constitutes a crucial process during local tissue invasion and metastasis [5]. By secreting proteolytic enzymes, tumor cells can create a path to migrate both locally and distantly. Matrix metalloproteinases (MMPs) belong to a family of zinc-dependent endopeptidases that degrade several components of the ECM [6]. The structure and substrate of the MMPs family allows it to be divided into subgroups of collagenases, stromelysins, gelatinases, membrane-type MMPs, and other MMPs [7]. These are crucial to normal physiological processes, such as embryonic development, inflammation, angiogenesis, and wound healing. However, recent research has demonstrated that high levels of MMPs are often correlated with human cancers, such as lung [8], breast [9], liver [10], and oral cancers [11]. The activities of MMPs are regulated by physiological inhibitors, tissue inhibitors of metalloproteinases (TIMPs) [12]. The imbalance of active MMPs and TIMPs is a crucial element involved in the remodeling of the ECM exhibited in a number of disease states [13]. Gohji et al. suggested that the serum MMP-2:TIMP-2 ratio is an independent prognostic indicator of urothelial cancer recurrence[14].

Flavonoids are polyphenolic compounds that are found in fruits and vegetables [15]. Flavonoids are commonly used in cardiovascular disease prevention [16], [17]. In addition, previous studies have demonstrated that dietary flavonoids inhibit the development of various human cancers, such as breast cancer [18], prostate cancer [19] and colorectal cancer [20]. Kaempferol, a natural polyphenol belonging to the flavonoid group, is present at high levels in tea, grapes, broccoli, and berries [21], [22]. Several previous studies have indicated that kaempferol exhibits antioxidant [23], anti-inflammation [24] and anti-tumor properties [25]. There are at least six different types of flavonoids. Kaempferol belongs to the flavonol and have a similar structure as quercein and myricetin, which also have anti-cancer effects [26]. Kaempferol was discovered to inhibit angiogenesis and VEGF expression in human ovarian cancer cells, and the pathways involved the regulation of HIF-1α [27]. Kaempferol also produced an apoptosis effect through AKT expression in human glioma cells [28] and leukemia cells [29]. Recent studies have indicated that kaempferol induces G2/M cell cycle arrest and autophagic cell death in human hepatic cancer cells [30]. Moreover, Kang et al., reported that kaempferol and quercetin induced caspase-3-dependent apoptosis in oral cavity cancer cells [31]. However, the effect of kaempferol on the cancer metastasis of OSCC, and the underlying mechanisms of this effect, has not yet been studied. In this study, we demonstrate that the suppression of metastatic ability by kaempferol is produced by the down-regulation of MMP-2 expression, and we hope to provide a foundation for further research.

## Materials and Methods

### Cell and cell culture

SCC-4, a human tongue squamous cell carcinoma cell line obtained from ATCC (Manassas, VA, USA) was cultured in Dulbecco's modified Eagle's medium supplemented with an equal volume of a nutrient mixture, F-12 Ham's medium (Life Technologies, Grand Island, NY, USA), 10% fetal bovine serum (Hyclone Laboratories, Logan, UT, USA), 2 mM glutamine, 100 U/mL penicillin, 100 µg/mL streptomycin, and 400 ng/mL hydrocortisone. All cell cultures were maintained at 37 °C in a humidified atmosphere of 5% CO_2_.

### Cell viability assay (MTT assay)

SCC4 cells were seeded in 24-well plates at a density of 5×10^4^ cells/well and treated with kaempferol at a concentration between 0–100 µM at 37°C for 24 h. After the exposure period, the media was removed, and cells were washed with phosphate buffered saline (PBS) and then incubated with 20 µL MTT (5 mg/mL) (Sigma chemical Co., St. Louis, MO, USA) for 4 h. The viable cell number per dish is directly proportional to the production of formazan, by dehydrogenases in the mitochondria within live cells, which can be measured spectrophotometrically at 563 nm following solubilization with isopropanol.

### Cell migration and invasion assays

After a treatment with kaempferol (0, 20, 40, 60, 80 and 100 µM) for 24 h, surviving cells were harvested and seeded to Boyden chamber (Neuro Probe, Cabin John, MD, USA) at 10^4^ cells/well in serum free medium and then incubated for 24 h or 48 h at 37°C in the migration assay or invasion assay, respectively. For invasion assay, 10 µL Matrigel (25 mg/50 mL; BD Biosciences, MA, USA) was applied to 8 µm pore size polycarbonate membrane filters and the bottom chamber contained standard medium. The invaded cells were fixed with 100% methanol and stained with 5% Giemsa. Cell numbers were counted under a light microscope. The migration assay was carried out as described in the invasion assay with no coating of Matrigel.

### Gelatin substrate gel zymography

The activities of MMP-2 in conditional medium were measured by gelatin zymography protease assays. Briefly, collected media of an appropriate volume (adjusted by vital cell number) were prepared with SDS sample buffer without boiling or reduction and subjected to 0.1% gelatin-8% SDS-PAGE electrophoresis. After electrophoresis, gels were washed with 2.5% Triton X-100 and then incubated in reaction buffer (40 mM Tris–HCl, pH 8.0; 10 mM CaCl_2_ and 0.01% NaN_3_) for 12 h at 37°C. Then gel was stained with Coomassie brilliant blue R-250. Bands corresponding to MMP-2 activity were visualized by negative staining using 0.3% Coomassie blue in 50% methanol and 10% acetic acid.

### Western blot analysis

Cellular lysates were prepared by suspending 2×10^6^/10 cm dish in 200 µL of RIPA buffer containing protease inhibitors cocktail. Cell lysates were subjected to a centrifugation of 10,000 rpm for 10 min at 4°C, and the insoluble pellet was discarded. The protein concentration of total cell lysates was determined by Bradford assay. The 20 µg samples of total cell lysates or nuclear fractions were separated by SDS-PAGE on 10% polyacrylamide gels and transferred onto a nitrocellulose membrane using the Mini-Protean Tetra Electrophoresis System as described previously [32]. The blot was subsequently incubated with 5% non-fat milk in Tris-buffered saline (20 mM Tris, 137 mM NaCl, pH 7.6) for 1 h to block non-specific binding and then overnight with polyclonal antibodies against MMP-2, TIMP-2 or three MAPKs (ERK 1/2, JNK 1/2 and p38) with the specific antibodies for unphosphorylated or phosphorylated forms of the corresponding ERK 1/2, JNK 1/2 and p38. Blots were then incubated with a horseradish peroxidase goat anti-rabbit or anti-mouse IgG for 1 h. Afterwards, signal was detected by using enhanced chemiluminescence (ECL) commercial kit (Amersham Biosciences) and relative photographic density was quantitated by scanning the photographic negatives on a gel documentation and analysis system (AlphaImager HP System, Alpha Innotech Corporation, San Leandro, CA, USA).

### RNA isolation, semi-quantitative RT-PCR and taqman quantitative real-time PCR

Total RNA was isolated from 1×10^6^ SCC4 cells using Trizol (Life Technologies, Grand Island, NY) according to the manufacturer's instructions. Total RNA (2 µg) was reverse transcribed into cDNA by SuperScript III First-Strand Synthesis Supermix (Invitrogen, Carlsbad, CA). The PCR was performed in a reaction mixture containing 2 µL cDNA, 0.2 mM dNTP mixture, 2 µM of each primers, 1 U Taq DNA polymerase, and 1-fold concentration of Thermal Pol Buffer (New England BioLabs, MA, USA) by denaturation at 95°C for 5 min, followed by amplification of indicated cycles of 95°C for 30 sec, 62°C for 30 sec, and 72°C for 30 sec. The specific primer sequences for these genes are as following: MMP-2: 5′-GGCCCTGTCACTCCTGAGAT-3′ (forward), 5′-GGCATCCAGGTTATCGGGG A-3′ (reverse), and TIMP-2: 5′-GGCGTTTTGCAATGCAGATGTAG-3′ (forward), 5′-CACAGGAGCCGTCACTTCTCTTG-3′ (reverse). Quantitative real-time PCR analysis was carried out using Taqman one-step PCR Master Mix (Applied Biosystems). 100 ng of total cDNA was added per 25 µl reaction with MMP-2 or GAPDH primers and Taqman probes. The MMP-2 and GAPDH primers and probes were designed using commercial software (ABI PRISM Sequence Detection System; Applied Biosystems). Quantitative real-time PCR assays were carried out in triplicate on a StepOnePlus sequence detection system. The threshold was set above the non-template control background and within the linear phase of target gene amplification to calculate the cycle number at which the transcript was detected.

### Luciferase reporter gene assay

SCC4 cells were seeded at a concentration of 5×10^4^ cells per well in 6-well cell culture plates. A fragment of the MMP-2 promoter was inserted into the pGL3-basic vector to generate the MMP-2 promoter plasmid. After 24 h of incubation, pGL3-basic (vector) and MMP-2 promoter plasmid were co-transfected with a β- galactosidase expression vector (pCH110) into cells using Turbofect (Fermentas, Carlsbad, CA). After 12 h of transfection, cells were treated with vehicle or kaempferol (0, 20, 40, 60, 80 and 100 µM) for 24 h. Luciferase and β-galactosidase activities were assayed according to the manufacturer's protocol (Promega). Luminescence was measured using a Tropix TR717 Microplate Luminometer (Applied Biosystems). The value of the luciferase activity was normalized to transfection efficiency and monitored by β-galactosidase expression.

### Electrophoretic mobility shift assay

AP-1 binding assays in nuclear extracts were performed with biotin-labeled double-stranded c-Jun oligonucleotides (5′-CGCTTGATGAGTCAGCCGGAA-3′), and the electrophoretic mobility shift assay was carried out by using the Lightshift kit (Promega). Briefly, binding reactions containing 10 µg of nuclear protein, 10 mM Tris, 50 mM KCl, 1 mM dithiothreitol, 5 mM MgCl_2_, 2 µg poly (dI·dC) and 2 pmole of oligonucleotide probe were incubated for 20 min at room temperature. Protein DNA complexes were separated by electrophoresis on a 6% non-denaturing acrylamide gel, transferred to positively charged nylon membranes and then cross-linked in a Stratagene cross-linker. Gel shifts were visualized with a streptavidin–horseradish peroxidase followed by chemiluminescent detection. The unlabeled oligos of AP-1 at 200× were added to compete specifically with labeled oligo binding in the competitive EMSA.

### Chromatin immunoprecipitation analysis (ChIP)

Chromatin immunoprecipitation analysis was performed as described previously [33]. In brief, chromatin and proteins from approximate 2×10^6^ cells were crosslinked with 1% formaldehyde for 10 min at room temperature. These cells were collected, lysed, and sonicated on ice to shear the chromatin DNA to a length between 200 – 1000 base pair by using Sonicator 3000 (Misonix, NY, USA). The sonicated chromatin lysate was immunoprecipitated with anti-c-Jun antibody, and collected with Protein A/G agarose beads (Pierce, IL, USA). The protein/DNA crosslinks of the immunoprecipitated complexes were reversed by incubation in 0.2 M NaCl at 65°C for 4 h, and then the DNA was purified and applied to PCR as described above to determine the binding ability of c-Jun to MMP-2 promoter. The sequences of the primers are 5′-CTCTTTAGCTCTTCAGGTCTCAGC-3′ (forward), and 5′-TGTTGGGAACGCCTGACT-3′ (reverse).

### Statistical analysis

For all of the measurements, analysis of variance followed by Scheffe posteriori comparison was used to assess the differences between control and cells treated with various concentration of kaempferol. A difference at p<0.05 was considered to be statistically significant and the experiments were repeated three times.

## Results

### Effect of kaempferol on the viability of SCC4 cells

**Figure 1:**
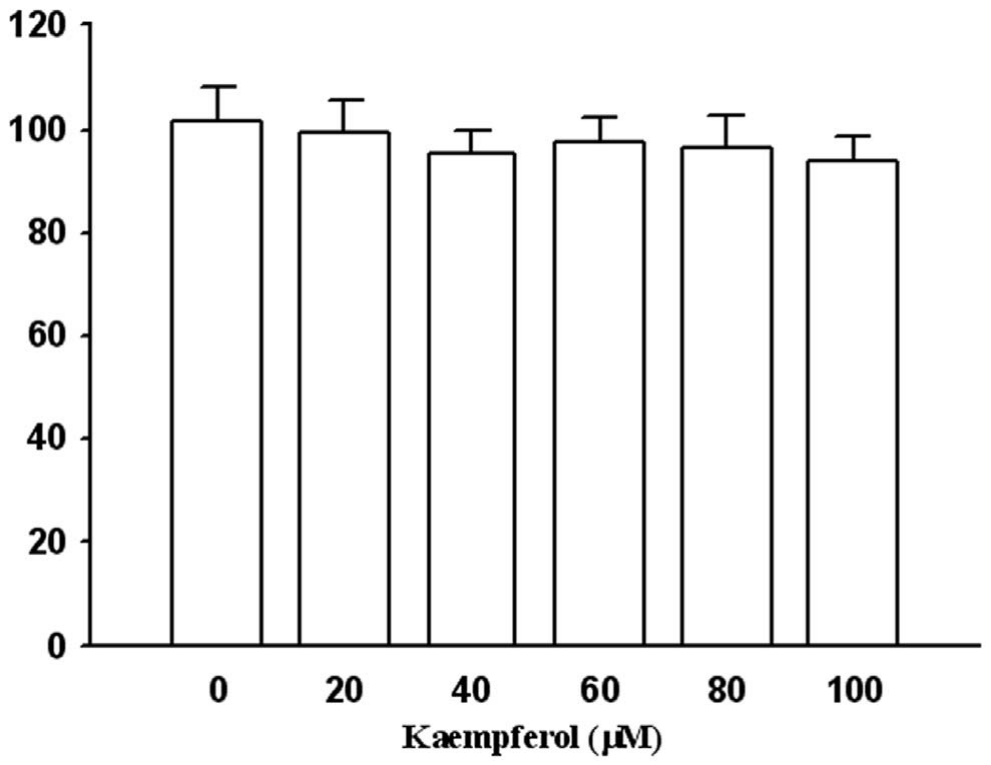
Effects of kaempferol on cell viability. SCC4 cells were treated with various concentrations (0, 20, 40, 60, 80, and 100 µM) of kaempferol for 24 hours. Cell viability was determined using an MTT assay. The values represented the means ± SD of at least three independent experiments.

We analyzed the cytotoxic effects of kaempferol at various concentrations (0–100 µM) on SCC4 cells by using the MTT assay. As shown in Figure 1, using kaempferol treatment on the SCC4 cells produced no cytotoxic effect on cell viability. Therefore, this concentration range of kaempferol was used in the following experiments.

### Kaempferol inhibits SCC4 cell migration and invasion

**Figure 2:**
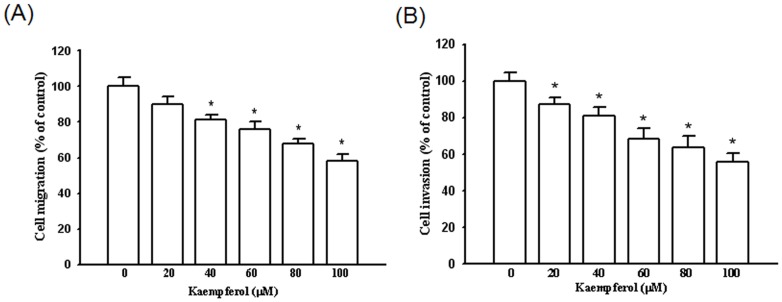
Effect of kaempferol on cell migration and invasion in SCC4 cells. After being treated with kaempferol at a concentration of 0, 20, 40, 60, 80, and 100 µM, (A) the cell migration and (B) cell invasion were measured using a Boyden chamber for 24 hours and 48 hours, respectively. The values represented the means ± SD of at least three independent experiments. *p<0.05 as compared with the control.

To examine whether the cell migration and invasion were suppressed by kaempferol, we seeded SCC4 cells in a Boyden chamber and calculated the number of invasive cells in the presence of kaempferol. We observed that kaempferol substantially inhibited the migration and invasion of SCC4 cells in a dose-dependent manner, with only 58% and 56% remaining after a treatment of 100 µM of kaempferol at 24 h and 48 h, respectively (Figures 2A and 2B).

### Kaempferol reduces the expression of MMP and TIMP

**Figure 3:**
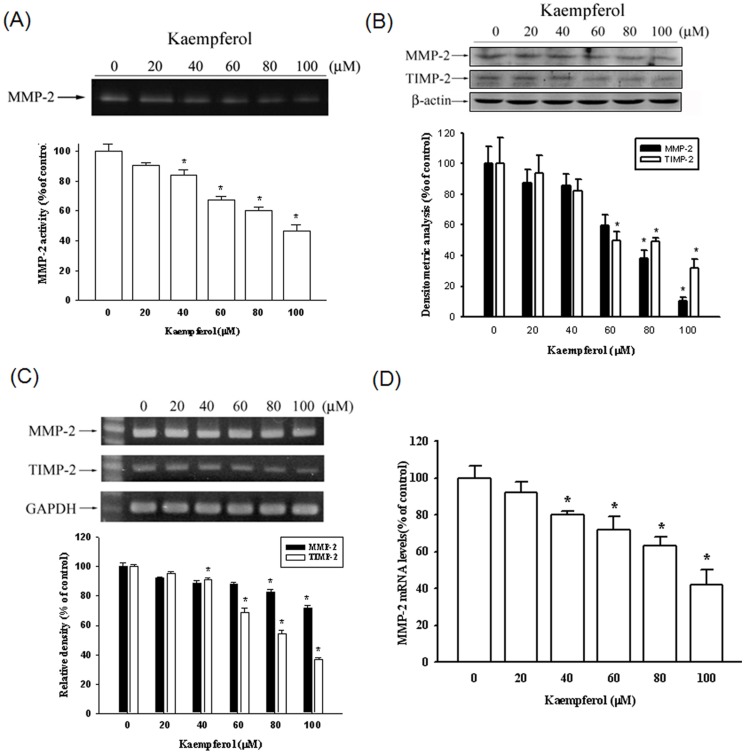
Effects of kaempferol on protein and mRNA levels of MMP-2 and the endogenous inhibitor TIMP-2. SCC4 cells were treated with various concentrations (0, 20, 40, 60, 80, and 100 µM) of kaempferol for 24 hours. The conditioned media were collected and the activity of MMP-2 was detected (A), or a western blot with anti-MMP-2 and anti-TIMP-2 antibodies was performed (B). (C) Semiquantitative RT-PCR was performed to compare MMP-2 and TIMP-2 mRNA levels. (D) The mRNA levels of MMP-2 were quantified using a real-time PCR assay. The values represented the means ± SD of at least three independent experiments. *p<0.05 as compared with the control.

To demonstrate whether the suppression of SCC4 migration was mediated by regulating the MMP-2 expression, a gelatin zymography assay was performed. The gelatin zymography data indicated that MMP-2 enzyme activity was inhibited by 53% at the highest concentration of kaempferol (100 µM) (Figure 3A). Figure 3B shows a Western blotting analysis of the MMP-2 and TIMP-2 protein levels. The protein levels of both MMP-2 and TIMP-2 decreased substantially. The mRNA expression also demonstrated the same results (Figure 3C). We also used a quantitative real-time PCR assay to confirm the MMP-2 mRNA expression (Figure 3D). Thus, Kaempferol considerably inhibits MMP-2 mRNA expression in a concentration-dependent manner.

### Kaempferol inhibits the transcriptional activity of MMP-2

**Figure 4:**
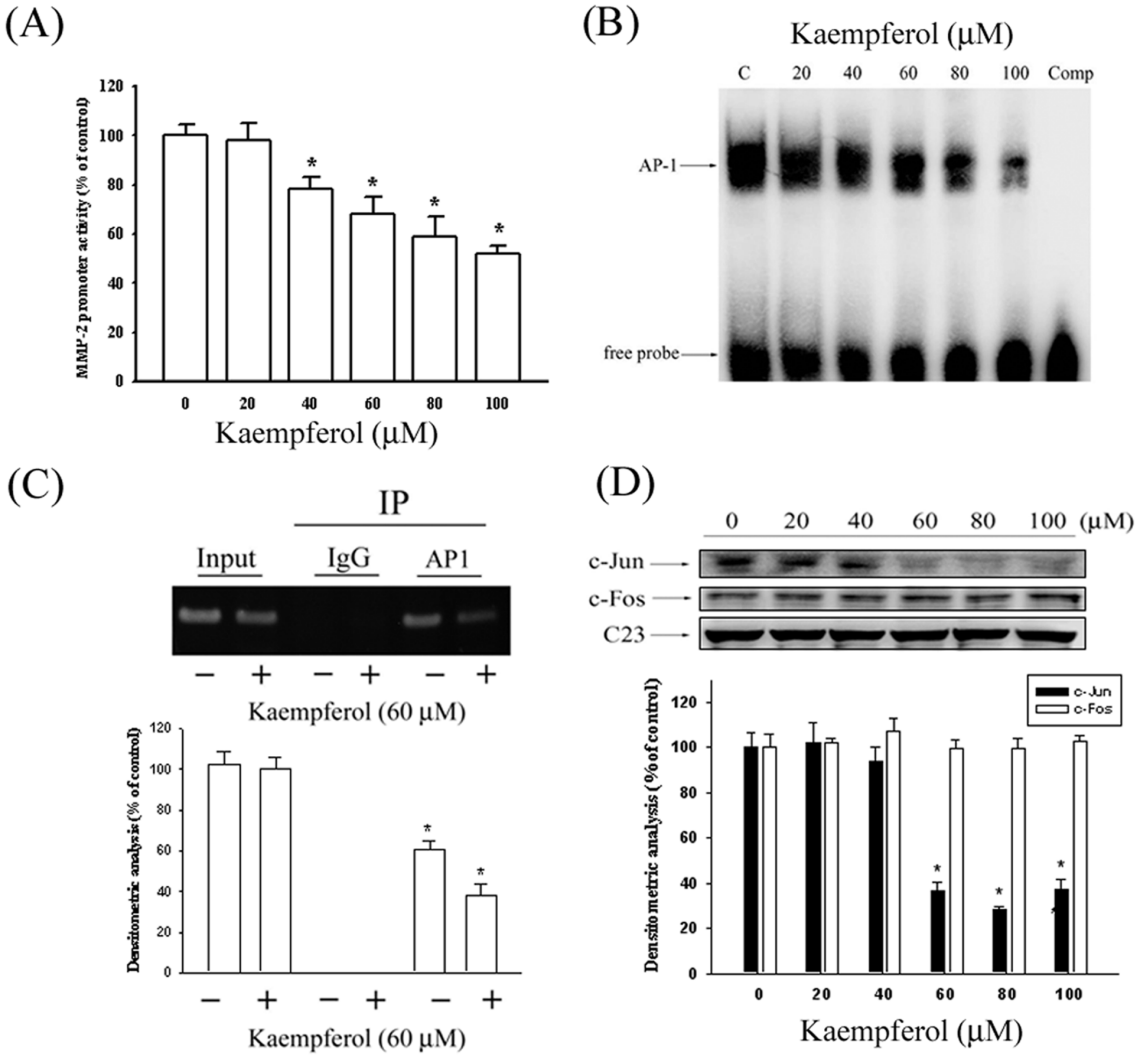
Kaempferol suppresses MMP-2 expression at a transcriptional level and on DNA binding activity of AP-1 in SCC4 cells. (A) SCC4 cells were transfected with pGL3 basic or a MMP-2 promoter/reporter plasmid, then treated with various concentrations (0, 20, 40, 60, 80, and 100 µM) of kaempferol. After 24-hour incubation, luciferase activities were determined and normalized to β-galactosidase activity. (B) Nuclear extract prepared from SCC4 cells with various concentrations (0, 20, 40, 60, 80, and 100 µM) of kaempferol were incubated with biotin-labeled AP-1-specific oligonucleotides with consensus sequence for AP-1 binding. Bound complexes were analyzed using an electrophoretic mobility shift assay. (C) Binding of c-Jun to the MMP-2 promoter was measured using a ChIP assay. (D) Representative results of c-Jun and c-Fos protein levels by using western blot analysis. The values represented the means ± SD of at least three independent experiments. *p<0.05 as compared with the control.

To further investigate whether the kaempferol regulated the promoter activity of MMP-2, we performed a luciferase reporter assay, and a reporter gene that contained the MMP-2 promoter region was transfected into the SCC4 cells. As shown in Figure 4A, the MMP-2 promoter activity was reduced in a dose-dependent manner, indicating that kaempferol inhibits MMP-2 expression at the transcriptional level.

### Kaempferol decreases c-Jun/AP-1 activation in SCC4 cells

Because previous data revealed that AP-1 was a key transcriptional regulator in MMP-2 promotion [33], EMSA and ChIP assays were then performed to investigate the effect of kaempferol on AP-1 DNA-binding activity. As illustrated in Figure 4B, the binding activity of AP-1 to the MMP-2 promoter decreased significantly in SCC4 cells after being treated with kaempferol in a concentration-dependent manner. Specifically, the binding capacity of AP-1 on the promoter of the MMP-2 gene was repressed in SCC4 cells after being treated with 60 µM kaempferol (Figure 4C).To determine the transcription factors, we used a Western blot assay to detect the nuclear translocation of c-Jun and c-Fos. Treating SCC4 cells with kaempferol reduced the nuclear translocation of c-Jun, but produced no effects on the level of c-Fos (Figure 4D).

### Kaempferol inhibits the phosphorylation of ERK1/2

**Figure 5:**
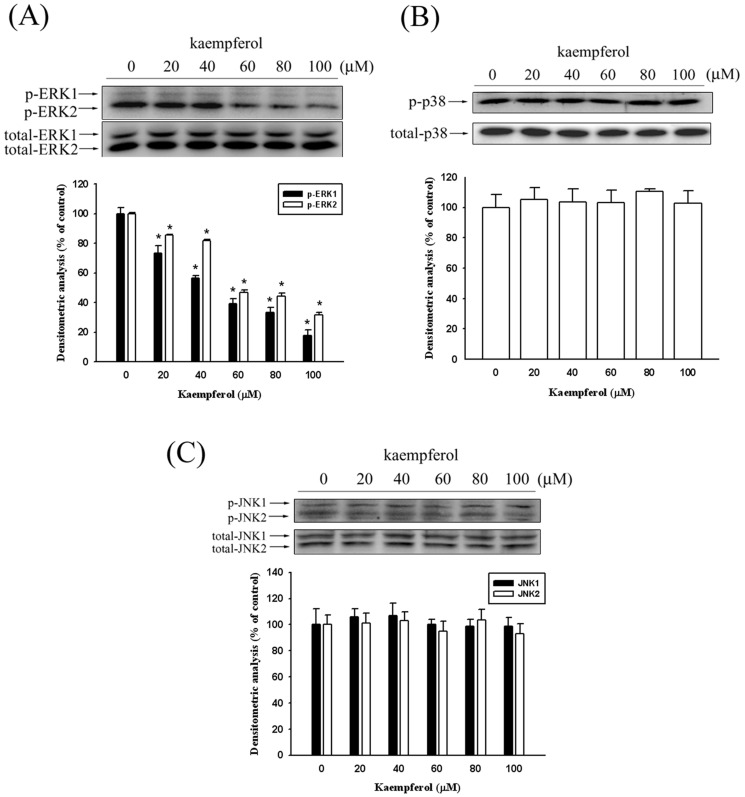
Effects of kaempferol on the signaling pathways. SCC4 cells were treated with various doses of kaempferol (0, 20, 40, 60, 80, and 100 µM) for 24 hours and whole cell lysates prepared from these cells were used for western blot analysis with (A) anti-ERK1/2, (B) anti-JNK1/2 and (C) anti-p38 (total and phosphorylated) antibodies as described in the Materials and Methods section. The values represented the means ± SD of at least three independent experiments. *p<0.05 as compared with the control.

According to the data we collected, kaempferol inhibited SCC4 cell migration by reducing the MMP-2 expression. To further investigate the underlying mechanism of the anti-metastatic ability of kaempferol in SCC4 cells, we used a Western blot assay to detect the expression of the MAPK pathways. Figure 5A shows that the phosphorylation of ERK was suppressed after treating the SCC4 cells with kaempferol. However, the phosphorylation of the JNK1/2 and p38 pathways remained unaffected (Figures 5B–5C).

### Effect of kaempferol on the MMP-2 expression of SCC4 cells treated with U0126

**Figure 6:**
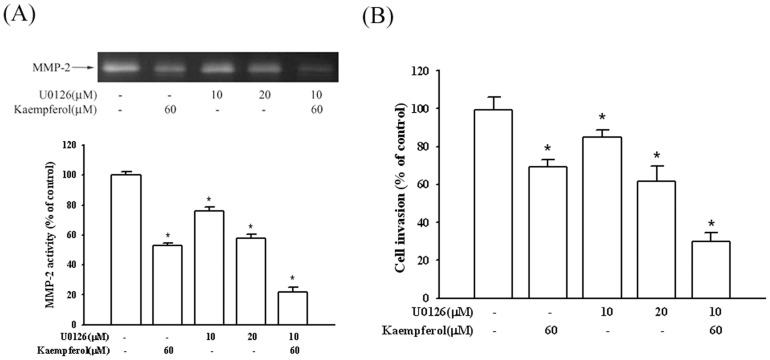
Effects of MEK inhibitor (U0126) and kaempferol on MMP-2 activity and invasion of SCC4 cells. SCC4 cells were pre-treated with U0126 (10 or 20 µM) for 1 hour, and then incubated in the presence or absence of kaempferol (60 µM) for 24 hours. (A) The culture media were used as subjects for analysis of MMP-2 activity, and cells were used for invasion assay (B) as described in the Materials and Methods section. The values represented the means ± SD of at least three independent experiments. *p<0.05 as compared with the control.

To demonstrate whether the suppression of the MMP-2 expression by kaempferol occurred mainly by inhibiting the ERK1/2 signaling pathway, SCC4 cells were treated with U0126, a MEK inhibitor. The gelatin zymography data demonstrated that MMP-2 enzyme activity was suppressed when only treating kaempferol or U0126 with 47% and 42%. However, combination treatment of the inhibitor with kaempferol intensively reduced MMP-2 enzyme activity by 78% (Figure 6A). In addition, similar results were obtained from the Boyden chamber assay of cellular invasion. Figure 6B shows that both kaempferol and U0126 inhibit cell invasion, and treatments combining these two chemical compounds enhance anti-invasion activity. Therefore, the inhibition of the ERK1/2 signaling pathways may result in a reduced expression of MMP-2, and reduced tumor cell migration.

## Discussion

The consumption of fruits and vegetables containing flavonoids produces vital health benefits. Kaempferol, a flavonoid, demonstrates diverse biological and pharmacological effects, such as antioxidant activity and anti-cancer-related properties [23], [31], [34]. Our study used SCC4 oral squamous cell carcinoma cells, and our results indicate that kaempferol (1) inhibits the migration and invasion of SCC4 cells; (2) reduces the gene expression and enzyme activity of MMP-2; (3) decreases the nuclear translocation of AP-1 to the MMP-2 promoter; and (4) inhibits the phosphorylation of ERK1/2.

Several previous studies have demonstrated that numerous phytochemicals use anti-metastasis abilities by suppressing the enzyme activity or gene expression of MMP-2 [35], [36]. Caffeic acid phenethyl ester inhibits oral cancer cell metastasis by regulating the MMP-2 and MAPK pathways [11]. Silibinin suppresses human osteosarcoma MG-63 cell invasion by inhibiting the ERK-dependent induction of MMP-2[37]. Wang et al observed that PAK5-Egr1-MMP-2 signaling controls the migration and invasion in breast cancer cells [38]. Although TIMP-2 was the physiological inhibitor of MMP-2, our data indicate that kaempferol reduces MMP-2 and TIMP-2 mRNA, and protein expression. In previous studies, TIMP-2 overexpression reduced invasion and angiogenesis, and protected melanoma cells from apoptosis [39]. Bourboulia et al. revealed that TIMP-2 promotes an anti-tumoral transcriptional profile by up-regulating E-cadherin in lung cancer cells [40]. However, de Vicente et al. demonstrated that the expression of TIMP-2 in oral squamous cell carcinoma was correlated with TNM staging, local recurrence, and less favorable survival rates [41]. The same findings were included in the study performed by Baker et al. who discovered higher average levels of TIMP-2 in tumor tissue than in normal tissue [42].

Previous studies have comprehensively demonstrated the role of the MAPK pathway in regulating MMPs expression [37], [43], [44]. Shan et al. indicated that estrogen can increase the expression of VEGF, and activate the ERK1/2 pathway to induce MMP-2 expression [45]. Silibinin inhibits the invasion of oral cancer cells by suppressing the activation of ERK1/2 and the MMP-2 expression [46]. Selaginella tamariscina (Beauv.), a traditional medicinal plant, possesses antimetastatic effects on human osteosarcoma cells by decreasing MMP-2 and MMP-9 secretions through p38 and Akt signaling pathways[47]. However, our study results showed that kaempferol only inhibits ERK phosphorylation and no significant effects were evident on the JNK, and p38 signaling pathways. Indeed, as shown in Figures 5–6, kaempferol inhibited the phosphorylation of ERK1/2, and the involvement of the MAPK pathway was well-demonstrated by using the MEK inhibitor in SCC4 cells, thus showing that a treatment using U0126 could lead to an inhibition of MMP-2 secretion and SCC4 cell invasion.

The expression of the MMP-2 gene is regulated by the transcriptional level interaction of AP-1 with binding sequences in the MMP-2 gene promoter [33], [48]. AP-1 is not a single transcription factor, but a heterodimer consisting of c-Fos and c-Jun families. Several reports have demonstrated that numerous drugs inhibit cancer metastasis by modulating the DNA-binding activities of AP-1. Hong et al. indicated that ascochlorin inhibits MMP-9 expression by suppressing AP-1 activity [49]. Nobiletin, a citrus flavonoid, attenuated MMP-7 expression by reducing AP-1 DNA-binding activity [50]. Our present data reveal that kaempferol decreased the MMP-2 activity of SCC4 cells by inhibiting AP-1 activation. This finding supports previous reports that indicated silibinin suppressed human osteosarcoma cell invasion by inhibiting the ERK-dependent AP-1 induction of MMP-2 [37]. However, we observed that kaempferol depresses only the c-Jun expression in the nucleus without affecting c-Fos. All of these results suggest that kaempferol inhibits SCC4 cell migration and invasion by decreasing the MMP-2 gene promoter binding activity of AP-1 transcription factors, including c-Jun.

In summary, our results indicate that kaempferol inhibits AP-1 activity, reduces MMP-2 expression, and subsequently suppresses the invasion of SCC4 cells. Furthermore, we demonstrated that kaempferol inhibits ERK1/2 phosphorylation, effectively leading to MMP-2 down-regulation. These results suggest that kaempferol may be a powerful candidate in the development of agents used for preventing cancer metastasis.
